# Ultrasound Microbubble-Mediated microRNA-505 Regulates Cervical Cancer Cell Growth via AKT2

**DOI:** 10.1155/2020/3731953

**Published:** 2020-10-15

**Authors:** Leilei Xu, Qin Zhang, Changhua Li, Fu Hua, Xiaoping Liu

**Affiliations:** ^1^Department of Gynecology, The First Hospital of Hua'an Affiliated to Nanjing Medical University, Huai'an, 223300 Jiangsu, China; ^2^Department of Ultrasound, The First Hospital of Huai'an Affiliated to Nanjing Medical University, Huai'an, 223300 Jiangsu, China

## Abstract

The application of ultrasound and microbubbles (USMB-) mediated microRNA (miR) is a promising approach of gene delivery for cancer treatment. We aimed to discuss the effects of USMB-miR-505 on cervical cancer (CC) development. miR-505 mediated by USMB was prepared. The effect of miR-505 on its transfection efficiency and the effect of miR-505 on HeLa cell proliferation, cell cycle, apoptosis, migration, and invasion were studied. The target gene of miR-505 was predicted, and its expression in CC was detected. The effect of the target gene on HeLa cells was further verified. USMB-miR-505 showed a higher transfection efficiency than miR-505 alone. The inhibitory effect of miR-505 mediated by USMB on HeLa cells was better than miR-505. miR-505 targeted AKT2, which was upregulated in CC. Overexpression of AKT2 reversed the inhibitory effect of USMB-miR-505 on HeLa cell malignant behaviors. Overall, we highlighted that USMB-miR-505 inhibited HeLa cell malignant behaviors by targeting AKT2.

## 1. Introduction

Cervical cancer (CC) is an aggressive gynecological malignancy with a high risk of recurrence and death, mainly in women [1, 2]. It is projected to be the 2nd most prevalent cancer in females aged 20-39 years in 2020 in the United States [3–5]. The incidence varies across the world, with the highest incidence rate in Eastern Africa and the lowest incidence in Western Asia [6]. Over 70% of CC cases diagnosed in developing countries are locally invasive or metastatic, and the diagnosis of early-stage CC is difficult, leading to high mortality [7]. Numerous efforts have been made to prevent CC that include the expansion of human papillomavirus vaccine coverage and primary screening [8], which eventually reduces the disease in many developed countries [9, 10]. Fortunately, radical hysterectomy has gained increasing popularity in the last twenty years and enjoyed broad acceptance across Europe and the Americas [11]. Although there is great progress in diagnostic and therapeutic strategies, the survival rate for CC patients remains poor [12]. Thus, this study aims to identify novel biomarkers and develop noninvasive therapies for the diagnosis and prognosis of CC.

microRNAs (miRs) participate in most biological processes like apoptosis and epithelial-to-mesenchymal transition (EMT) by regulating gene expression [13]. miRs and genes can serve as biomarkers for prognosis and treatment of CC [14]. miR-505 suppressed EMT and metastasis phenotypes in HK-1 cells and prevented macroscopic lung metastases [13]. But the role of miR-505 in CC is less studied. Microbubbles (MBs) were originally developed for ultrasound imaging, and now they are considered as ultrasound-assisted gene delivery tools to disturb cell membranes and accelerate gene entering into cells [15]. Behaviors of MBs under ultrasonic irradiation will lead to short-term membrane permeability of surrounding cells, thus promoting targeted local administration without cell damage [16]. The combination of ultrasound and microbubbles (USMB) is an emerging approach for noninvasive enhancement of uptake of drugs and genes [17, 18]. Microbubbles promote ultrasound-mediated gene transfer efficiency in cell culture and tumor transplantation of hindlimb and may selectively transfer therapeutic genes to disease sites [19]. USMB-mediated miR (USMB-miR) delivery has been considered as an effective tool for the treatment of cancer and cardiovascular diseases [20, 21]. Interestingly, USMB-miR-133a prevented tumor growth and improved survival of breast cancer in mice. USMB-mediated miR-767 silencing offered a novel therapeutic strategy for non-small-cell lung cancer treatment [22, 23]. However, the mechanism of USMB-miR-505 in CC remains unclear. We performed serials of molecular and histological experiments to evaluate the relevance of USMB-miR-505 in CC development.

## 2. Materials and Methods

### 2.1. Ethics Statement

The study followed the *Declaration of Helsinki* and was approved by the ethics committee of the First Hospital of Huai'an Affiliated to Nanjing Medical University. All patients signed the informed consent.

### 2.2. Sample Collection

From March 2018 to January 2019, cancer and adjacent normal tissues of 20 CC patients undergoing surgery in the First Hospital of Huai'an Affiliated to Nanjing Medical University were collected. The patients aged between 45 and 69 years old. They were free of any other malignant tumors and did not receive preoperative radiotherapy and chemotherapy. The tissues were immediately preserved at -80°C.

### 2.3. Cell Culture

HeLa cells at logarithm phase from the Cell Bank of Chinese Academy of Sciences (Shanghai, China) were cultured at 37°C with 5% CO_2_ in Dulbecco's modified Eagle medium (DMEM, Gibco; Waltham, MA, USA) with 10% fetal bovine serum (FBS, Gemini Bio-products, West-Sacramento, CA, USA) and 1% penicillin/streptomycin (Invitrogen, Carlsbad, CA, USA). The media were renewed every 2 days. Cells were subcultured when reached 80% confluence.

### 2.4. Cell Transfection and Grouping

miR-505 mimic, overexpression (OE)-AKT2, and their respective negative controls (NC) were designed and synthesized by GenePharma (Shanghai, China), while miR-505 mediated by USMB was prepared in the laboratory. HeLa cells were assigned into NC mimic, miR-505 mimic, miR-505-MB, miR-505-MB+OE-NC, and miR-505-MB+overexpression-AKT2 groups.

NC mimic or miR-505 mimic (1 *μ*g) was mixed with 2 *μ*L Lipofectamine 2000 (Invitrogen, Carlsbad, CA, USA). Then, cells were suspended again with 500 *μ*L DMEM. The mixed solution was put into the cells at 50 *μ*L/well. Next, cells in the miR-505-MB group were treated with miR-505-MB at 50 *μ*L/well and subjected to 10 MHz ultrasound for 30 minutes. Based on the treatment in the miR-505-MB group, cells in the miR-505-MB+OE-NC or miR-505-MB+OE-AKT2 group were stably transfected with corresponding vectors using Lipofectamine 2000 after the miR-505 expression was detected. After transfection and ultrasound exposure, cells were harvested for the following experiments.

### 2.5. Preparation of USMB

The USMB was prepared as previously described [24]. MBs were synthesized by ultrasonic dispersion of 1 mg/mL polyethylene glycol-40 stearate, 2 mg/mL 1-bisstearoyl phosphatidylcholine, 0.4 mg/mL 1,2-bisstearoyl-3-trifluoromethylpropane, and decafluorobutane (Avanti Polar Lipids Inc., Alabaster, AL, USA) in a water box. After that, the MBs were observed under an inverted fluorescence microscope (DM 4000B, Leica, Germany) and detected by a nanometer particle size analyzer (NS-90, OMEC Instruments Co., Ltd., Zhuhai, Guangdong, China). The MBs were filtered using a 1 *μ*m filtration membrane and adjusted into 0.8-1.6 × 10^9^/mL. Thereafter, 1 *μ*g miR-505 mimic was blended with 50 *μ*L MBs suspension and cultured at 37°C for 30 minutes. The unbounded miR-505 was removed by 0.16 M phosphate buffer saline (PBS) to harvest miR-505-MB.

### 2.6. Reverse Transcription Quantitative Polymerase Chain Reaction (RT-qPCR)

Total RNA was extracted by TRIzol (Invitrogen, Carlsbad, CA, USA) and reversely transcribed into cDNA using a cDNA reverse transcription kit (TOYOBO, Japan). The expression of message RNAs (mRNAs) and miR was quantified using a 7500 Fast Real-Time PCR System (Applied Biosystems, USA) with a SYBR green PCR Master Mix (TOYOBO, Japan), with U6 or GAPDH as references. The expression was calculated by the 2^−*ΔΔ*Ct^ method. The primers used are presented in Table 1.

### 2.7. 3-(4,5-Dimethylthiazol-2-yl)-2,5-Diphenyltetrazolium Bromide (MTT) Assay

An MTT kit (C0009, Beyotime, Shanghai, China) was employed to examine the viability of cells. In each well was added 10 *μ*L MTT solution, and the cells were incubated for 4 hours. Then, it was added 100 *μ*L solubilization solution (Formazan solvent) and mixed properly. After incubation at 37°C for 3-4 hours, the optical density (OD) value at 570 nm was determined with a microplate reader (Bio-Rad, Inc., Hercules, CA, USA).

### 2.8. Colony Formation Assay

After 24 hours of transfection, 500 cells were seeded into the 6-well plates and cultured for two weeks. The cell colonies were fixed for 5 minutes with methanol and stained for 15 minutes with 0.1% crystal violet. Thereafter, cell colonies were counted and photographed.

### 2.9. Flow Cytometry

Flow cytometry was utilized to detect cell cycles. After 48 hours of transfection, the cell cycle was evaluated by propidium iodide (PI) staining. Then, the cells were detached with trypsin, fixed at 4°C overnight in 70% cold ethanol, and reacted with 50 *μ*g/mL PI (KeyGene, Rockville, MD, USA) for 30 minutes. Cell cycle analysis was performed immediately using a FACS Calibur flow cytometer (BD, San Diego, USA). The proportion of cells in G1, S, and G2 phases was detected.

### 2.10. Enzyme-Linked Immunosorbent Assay (ELISA)

Human Bax (ab199080) and Bcl-2 (ab119506) were measured according to the ELISA kits (Abcam, Cambridge, MA, USA). Briefly, the cell extract, 1× wash buffer PT, and antibody cocktail were prepared according to the manufacturer's protocol. In a 96-well plate, the sample (50 *μ*L) was incubated with 50 *μ*L antibody cocktail for 1 hour at room temperature. The cells were then washed three times with 350 *μ*L 1× wash buffer PT and incubated with 100 *μ*L tetramethylbenzidine development solution for 15 minutes. After the termination of the reaction with 100 *μ*L stop solution, the OD value at 450 nm was read using a spectrophotometer (Lab-spectrum, Shanghai, China). The standard curve of target proteins was plotted, and the relative expression of target proteins in each group of cells was measured.

### 2.11. Hoechst 33258 Staining

Hoechst staining was used to detect cell apoptosis. As cells undergo apoptosis, chromatin sequestrates. After Hoechst staining, the nuclei of normal cells appear normal blue, while the nuclei of apoptotic cells are abnormally bright or whitish in color. The transfected cells were seeded at 1 × 10^5^ cells/mL (3 mL per well) into 6-well plates. After the cells were incubated at 37°C with 5% CO_2_ for 24 hours, the media was removed. The cells were then fixed with paraformaldehyde, washed with PBS, and stained with Hoechst 33258 (HY-15558, MedChemExpress, NJ, USA) in the dark. After 30 minutes, the cells were observed under a fluorescence microscope, and 5 fields were randomly chosen from each slide for photograph. The apoptosis rate was calculated according to the formula: apoptosis rate = number of apoptotic cells/total number of cells × 100%.

### 2.12. Wound Healing Assay

The cells were plated into 6-well plates and cultured for 24 hours. Then, a 200 *μ*L pipette tip was applied to scratch 3 parallel lines, and the cells were washed twice in PBS. Then, the cells were cultivated at 37°C and photographed at 0 and 24 hours under a FSX100 microscope (Olympus Optical Co., Ltd., Tokyo, Japan) after wounding. The migration ratio was estimated by examining the change of the scratch area.

### 2.13. Transwell Assay

The invasion experiments were carried out in the Transwell chamber coated with Matrigel (BD Biosciences, San Jose, CA, USA). Briefly, 500 *μ*L DMEM with 10% FBS was supplemented to the basolateral chamber, while 2 × 10^5^ cells were paved in the apical chamber. After 24 hours, cells passing through the Matrigel were fixed with methanol and stained by 0.1% crystal violet, otherwise removed by cotton swabs. Finally, cells were counted and the stained cells represented the invasiveness.

### 2.14. Western Blot

Total protein was extracted using RIPA buffer (Solarbio Science & Technology Co., Ltd., Beijing, China) containing proteinase inhibitor. Then, the protein was quantified by a BCA kit (Thermo Fisher Scientific Inc., Waltham, MA, USA), separated using SDS-PAGE, transferred onto PVDF membranes (Millipore, Billerica, MA, USA), and sealed with 5% skim milk. Subsequently, the membranes were incubated with rabbit antihuman AKT2 (1 : 1000, #2964, Cell Signaling Technologies, Beverly, MA, USA) at 4°C overnight, with rabbit antihuman GAPDH (1 : 10000, ab181602, Abcam, Cambridge, MA, USA) as the control. Then, the membranes were probed with secondary antibody goat antirabbit IgG H&L (HRP, 1 : 25000, ab205718, Abcam, Cambridge, MA, USA) in the dark at 37°C for 1 hour. Finally, the immunoblots were subjected to enhanced chemiluminescence reagent (Millipore, Billerica, MA, USA).

### 2.15. Dual-Luciferase Reporter Gene Assay

The binding site between miR-505 and AKT2 was predicted from Starbase (http://starbase.sysu.edu.cn/) and amplified using PCR and cloned into pGL3 vector (Promega, Madison, WI, USA) to obtain AKT2 wild-type (WT). AKT2 mutant (MT) was obtained by mutating the binding site. These vectors were cotransfected with miR-505 mimic and its control into 293T cells (American Type Culture Collection, Manassas, VA, USA) by Lipofectamine 2000. After 48 hours of transfection, the luciferase activity was measured by the luciferase reporter system (Promega, Madison, WI, USA).

### 2.16. RNA Immunoprecipitation (RIP)

RIP lysis buffer kit (Millipore, Billerica, MA, USA) was used for RIP experiments. Summing up, HeLa cells were lysed in RIP lysis buffer, and RNA was precipitated with anti-AGO2 (Millipore, Billerica, MA, USA) and anti-IgG (Millipore, Billerica, MA, USA). TRIzol reagent was used to purify the immunoprecipitated RNA, and RT-qPCR was used to detect the gene expression.

### 2.17. Statistical Analysis

SPSS 22.0 (IBM Corp. Armonk, NY, USA) was used for data analysis. The obtained data of 3 independent experiments were described as mean ± standard deviation. Comparisons between group pairs were analyzed using a paired *t* test or unpaired *t* test, while comparisons among multigroups were analyzed through one-way or two-way analysis of variance (ANOVA). A pairwise comparison after ANOVA analysis was conducted using Tukey's multiple comparisons test. The *p* value shall be based on a two-sided test. *p* < 0.05 was considered as a statistical difference.

## 3. Results

### 3.1. USMB-miR-505 Promoted miR-505 Transfection Efficiency in HeLa Cells

**Figure 1:**
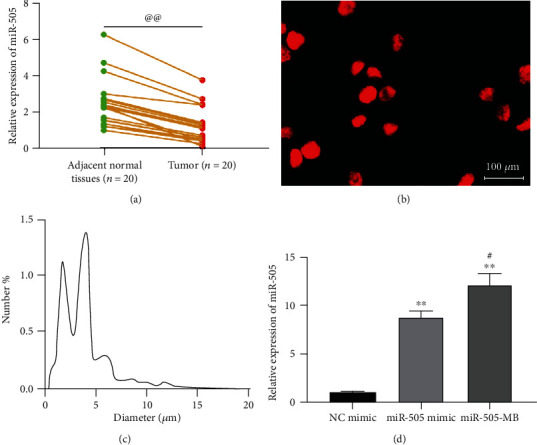
USMB enhanced the transfection efficiency of the miR-505 mimic. (a), miR-505 expression in CC and adjacent normal tissues detected by RT-qPCR. (b) The morphology of MBs observed under the inverted microscope. (c) The particle size of the prepared MBs determined by nanoparticle size analyzer. (d) The transfection efficiency measured by RT-qPCR. Paired *t* test for panel (a), ^@@^*p* < 0.01 vs. the adjacent normal tissues. One-way ANOVA for panel (d), ^∗∗^*p* < 0.01 vs. NC mimic, ^#^*p* < 0.05 vs. miR-505 mimic.

According to a previous report [25], miR-505 is poorly expressed in CC, which can inhibit the development of CC. The poor expression of miR-505 in CC was confirmed by RT-qPCR in our collected CC tissues (Figure 1(a)). The MBs were opalescent suspension, and the homogeneous parts were spherical and distributed uniformly (Figure 1(b)). The average particle size was about 2.16-4.68 *μ*m (Figure 1(c)).

RT-qPCR measured miR-505 expression in HeLa cells transfected with miR-505 mimic and miR-505-MB, which showed that the miR-505 mimic significantly increased miR-505 expression in HeLa cells, while the upregulation was more pronounced by USMB-miR-505 (Figure 1(d)).

### 3.2. USMB-miR-505 Suppressed the Proliferation and Promoted the Apoptosis of HeLa Cells

**Figure 2:**
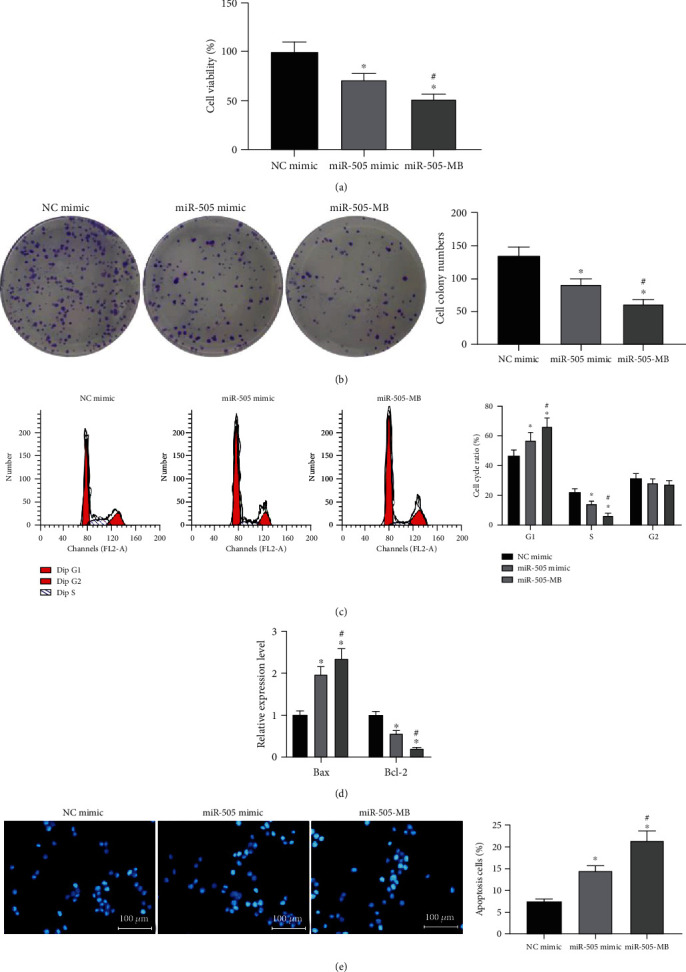
USMB-mediated miR-505 inhibited HeLa cell growth. (a) HeLa cell viability detected by MTT assay. (b) The ability of cell proliferation measured by colony formation assay. (c) Cell cycle detected by flow cytometry. (d) Bax and Bcl-2 expression detected by ELISA. (e) Hoechst staining detected the apoptosis rate. One-way ANOVA for data analysis in panels (a), (b), and (e). Two-way ANOVA for data analysis in panels (c) and (d). ^∗^*p* < 0.05 vs. NC mimic; ^#^*p* < 0.05 vs. miR-505 mimic.

The MTT assay and colony formation assays examined cell proliferation. The results demonstrated that the miR-505 mimic inhibited HeLa cell proliferation, while miR-505-MB strengthened the inhibitory effects of the miR-505 mimic (Figures 2(a) and (b)). Flow cytometry detected cell cycle changes in HeLa cells (Figure 2(c), Table 2) and found that the overexpression of miR-505 resulted in the increase of the G1 phase and the decrease of the S phase, especially in the miB-505-MB group. The expression of Bax and Bcl-2 in HeLa cells was measured by ELISA kits (Figure 2(d)). Compared with the NC mimic group, the Bax expression in HeLa cells of the other two groups increased and Bcl-2 expression decreased significantly, and the miR-505-MB group showed more powerful effects in regulating the Bcl-2 and Bax expression. Hoechst staining revealed that the overexpression of miR-505 led to the increase of the apoptosis rate of HeLa cells, especially in the miR-505-MB group (Figure 2(e)).

### 3.3. USMB-miR-505 Inhibited Migration, Invasion, and EMT of HeLa Cells

**Figure 3:**
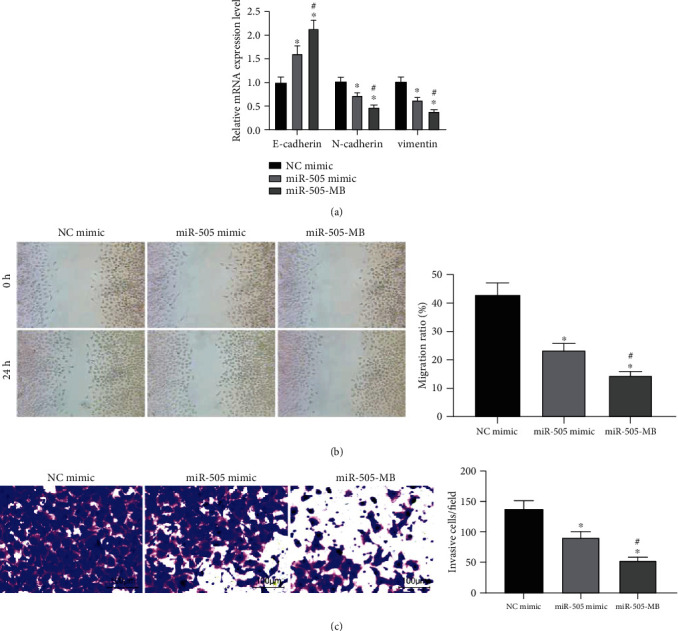
USMB-mediated miR-505 inhibited on HeLa cell migration and invasion. (a) Expression of EMT-related factors in HeLa cells measured by RT-qPCR. (b) The migration ability of cells measured by wound healing. (c) The invasion of cells examined by Transwell assay. One-way ANOVA for data analysis in panels (b) and (c). Two-way ANOVA for data analysis in panel (a). ^∗^*p* < 0.05 vs. NC mimic; ^#^*p* < 0.05 vs. miR-505 mimic.

In HeLa cells transfected with NC mimic, miR-505 mimic, and miR-505-MB, the expression of EMT-related factors E-cadherin, N-cadherin, and Vimentin was measured by RT-qPCR. The overexpression of miR-505, especially miR-505-MB, led to the increase of E-cadherin and the decrease of N-cadherin and Vimentin in HeLa cells (Figure 3(a)).

After 24 hours of wound healing and Transwell assays, the overexpression of miR-505 inhibited migration and invasion of HeLa cells, and the inhibitory effect of USMB-miR-505 was more pronounced (Figures 3(b) and (c)).

### 3.4. miR-505 Targets AKT2

**Figure 4:**
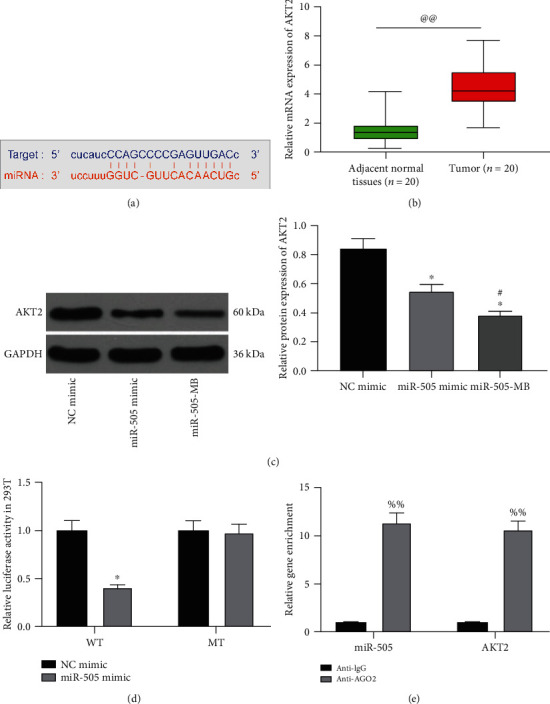
miR-505 targeted AKT2. (a) A binding site between miR-505 and AKT2. (b) AKT2 expression in CC and adjacent normal tissues detected by RT-qPCR. (c) WB detected AKT2 expression in HeLa cells. (d) The activity of WT-AKT2 and MT-AKT2 luciferase detected by dual-luciferase assay. (e) The binding relationship between miR-505 and AKT2 verified by the RIP experiment. Paired *t* test for panel (b), ^@@^*p* < 0.01 vs. the adjacent normal tissues; one-way ANOVA for data analysis in panel (c); two-way ANOVA for data analysis in panels (d) and (e); ^∗^*p* < 0.05 vs. NC mimic; ^#^*p* < 0.05 vs. miR-505 mimic; ^%%^*p* < 0.01 vs. anti-IgG.

Starbase (http://starbase.sysu.edu.cn/) predicted that miR-505 targeted AKT2 (Figure 4(a)). The expression of AKT2 in CC and adjacent normal tissues was examined by RT-qPCR, and AKT2 was significantly increased in CC tissues (Figure 4(b)). The AKT2 expression was measured by western blot in HeLa cells transfected with NC mimic, miR-505 mimic, and miR-505-MB. Compared with the NC mimic, the AKT2 expression in HeLa cells of the other two groups decreased significantly, especially in the miR-505-MB group (Figure 4(c)).

AKT2-WT and AKT2-MT were transfected with NC mimic or miR-505 mimic into 293T cells. The miR-505 mimic significantly reduced the luciferase activity of the AKT2-WT luciferase vector, but had no significant effect on the AKT2-MT luciferase vector (Figure 4(d)). The RIP experiment found that anti-AGO2 significantly enriched miR-505 and AKT2 (Figure 4(e)) compared with anti-IgG, which proved that miR-505 targeted AKT2.

### 3.5. Overexpression of AKT2 Compromised the Inhibitory Effects of USMB-miR-505 on HeLa Cells

In the HeLa cells transfected with miR-505-MB+OE-NC and miR-505-MB+OE-AKT2, the effect of overexpression of AKT2 on the HeLa cells treated with miR-505-MB was studied.

**Figure 5:**
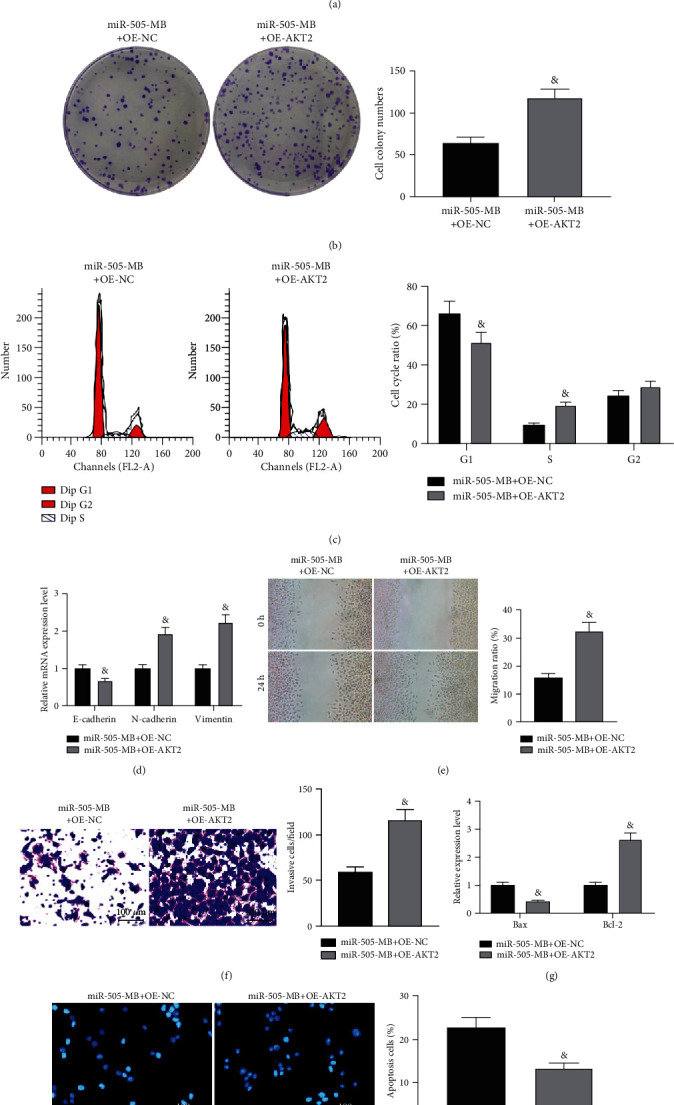
Overexpression of AKT2 reversed the effect of miR-505 mediated by USMB on HeLa cells. (a) MTT measured HeLa cell viability. (b) HeLa cell proliferation measured by colony formation assay. (c) Cell cycle changes measured by flow cytometry. (d) Expression of EMT-related factors measured by RT-qPCR. (e). HeLa cell migration was measured by wound healing. (f) HeLa cell invasion measured by Transwell assay. (g) Expression of apoptosis-related factors measured by ELISA. (h) Hoechst staining detected the apoptosis rate. Paired *t* test for panels (a), (b), (e), (f) and (h). ^&^*p* < 0.05 vs. the miR-505-MB+OE-NC group; two-way ANOVA for data analysis in panes (c), (d), and (g), ^&^*p* < 0.05 vs. the miR-505-MB+OE-NC group.

MTT and colony formation assays showed that the viability of HeLa cells treated with miR-505-MB elevated after the AKT2 overexpression (Figure 5(a)), and the number of cell colonies increased (Figure 5(b)). Flow cytometry showed that the overexpression of AKT2 attenuated the effect of miR-505-MB on HeLa cell cycle arrest (Figure 5(c), Table 3). RT-qPCR showed that the overexpression of AKT2 resulted in the decrease of E-cadherin and Bax and the increase of N-cadherin, Vimentin, and Bcl-2 in miR-505-MB-treated HeLa cells (Figure 5(d)). Wound healing and Transwell assays discovered that overexpression of AKT2 promoted migration and invasion of HeLa cells (Figures 5(e) and (f)). ELISA found that the overexpression of AKT2 resulted in the decrease of Bax and the increase of Bcl-2 (Figure 5(g)). Hoechst staining confirmed the inhibition of AKT2 on HeLa cell apoptosis (Figure 5(h)).

## 4. Discussion

**Figure 6:**
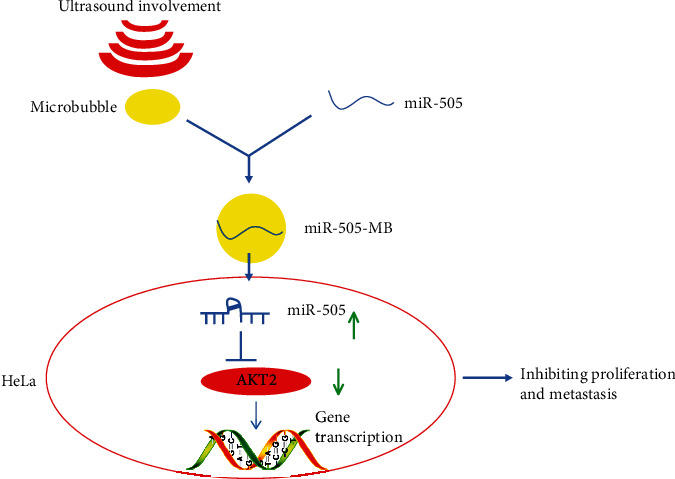
Experimental mechanism diagram. USMB-mediated miR-505 improves the transfection efficiency of miR-505 and inhibits the growth and metastasis of HeLa cells by targeting AKT2.

About 30%-35% of CC patients fail to completely recover from the treatment regimens, including surgical resection and radiotherapy [26]. Additionally, the conventional clinical variables, such as parametrial involvement and lymph node metastasis, are not enough to predict the curative effect or formulate the supplementary therapy for CC patients after surgery [27]. The combination of drug-loaded USMB has been used in preclinical studies on drug and gene delivery to solid tumors and the ablation of blood vessels [28]. Based on these facts, we discussed the possible mechanism of USMB-miR-505 in the malignant episodes of CC. As expected, our results provided evidence that USMB-miR-505 further strengthened the inhibitory role of miR-505 in CC malignancy by targeting AKT2 (Figure 6), which offered novel insights for CC treatment.

The abnormal expression of miRNAs is related to the pathological changes of humans, including cancer, and some are regarded as potential prognostic markers in different tumors, such as CC [29]. miR-505 was poorly expressed in CC and negatively correlated with tumor histology grade and lymph node metastasis [25, 30]. Additionally, miR-940 inhibited CC cell proliferation, and USMB-miR-940 showed better effects [31]. However, the role of the USMB treatment on miR-505 expression was not yet studied. MBs were synthesized by ultrasonic dispersion, and HeLa cells were transfected with USMB-mediated miR-505. We discovered that USMB-miR-505 had the most significant effect on miR-505 expression.

miRNAs can intervene in several aspects of CC, including proliferation, EMT, and chemosensitivity [32]. A recent study unveiled that paclitaxel-miR-34a-USMBs are a promising anticancer strategy for treating CC [21]. In this study, miR-505 blocked malignant episodes of HeLa cells, as demonstrated by higher Bax expression and lower Bcl-2 expression, which were reinforced by the USMB treatment. In line with our findings, the miR-505 inhibitor apparently elevated the cell viability, the colony numbers, and the migration rate, while downregulating the apoptotic rate and Bax expression in renal carcinoma cells [33]. Also, the overexpression of miR-505 induced the Bax expression in endometrial cancer cells, representing as a possible tumor suppressor [34]. The EMT is a crucial mechanism of tumor cell invasion and cancer metastasis, by which epithelial cells obtain mesenchymal fibroblast-like properties [35]. Vimentin is a well-recognized metastasis marker [36], while E-cadherin could repress the invasion and metastasis of epithelial cells [37]. EMT is a great contributor to the CC cell chemotherapy and radiotherapy resistance; thus, inhibiting EMT improves the survival rate of CC patients by sensitizing the CC cells to radiotherapy and drugs [32]. The miR-505 mimic elevated N-cadherin expression but decreased E-cadherin in gastric cancer cells [38]. miR-505-5p was correlated with metastasis, and the overexpression of miR-505-5p inhibited metastasis and EMT in CC cells [39]. In Ca-Ski and HeLa cells, the miR-505 upregulation suppressed the proliferation and tumorigenicity, indicating miR-505 may act as an inhibitor in CC [30]. Our study may offer a novel approach for CC treatment from the miR-505 delivery by USMB treatment.

Furthermore, miR-505 targeted AKT2. There are three subtypes of AKT, namely AKT1, AKT2, and AKT3, among which AKT2 is responsible for tumor progression and metastasis through regulating EMT-related proteins [40]. Increased AKT2 expression was associated with the cervical lesion progression [41]. AKT2 was highly expressed in CC tissues, and the AKT2 overexpression promoted the proliferation and colony formation ability in SiHa cells [42]. The overexpression of AKT2 was found to compromise the inhibitory effects of USMB-miR-505 on HeLa cells in the current study. It is known that activated AKT can promote the proliferation of cancer cells including CC [43]. The AKT2 expression was required for EMT-like morphological changes [44]. The knockdown of AKT2 prevented cell proliferation and stimulated apoptosis in CC cells [45]. A recent study showed that AKT2 inhibition has the potential for anticancer therapies for its function in EMT reversion, metastasis reduction, and tumor recurrence prevention in breast cancer [46]. To sum up, AKT2 silencing is possible to prevent CC development.

## 5. Conclusion

In conclusion, our study provided substantial evidence that the miR-505 overexpression repressed the CC progression, and USMB-mediated miR-505 reinforced the inhibitory effects of miR-505 in CC. These results indeed unveiled a promising approach for CC treatment. More works should be done in the future to identify the application value of our results. We will also figure out the possible downstream pathways involving the beneficial roles of USMB-miR-505 in CC treatment.

## Figures and Tables

**Table 1:** Primer sequences used for RT-qPCR.

Gene	Forward primer (5′-3′)	Reverse primer (5′-3′)
miR-505	GGAGCCAGGAAGTATTG	GAACATGTCTGCGTATCTC
AKT2	CATCCTCATGGAAGAGATCCGC	GAGGAAGAACCTGTGCTCCATG
E-cadherin	GCCTCCTGAAAAGAGAGTGGAAG	TGGCAGTGTCTCTCCAAATCCG
N-cadherin	CCTCCAGAGTTTACTGCCATGAC	GTAGGATCTCCGCCACTGATTC
Vimentin	AGGCAAAGCAGGAGTCCACTGA	ATCTGGCGTTCCAGGGACTCAT
GAPDH	GTCTCCTCTGACTTCAACAGCG	ACCACCCTGTTGCTGTAGCCAA
U6	ATTGGAACGATACAGAGAAGATT	AGGAACGCTTCACGAATTTG

RT-qPCR: reverse transcription quantitative polymerase chain reaction; miR-505: microRNA-505; GAPDH: glyceraldehyde-3-phosphate dehydrogenase.

**Table 2:** The relationship between the miR-505 expression and cell cycle changes.

Cell cycle	NC mimic	miR-505 mimic	miR-505-MB	*p* value (miR-505 mimic vs. NC mimic)	*p* value (miR-505-MB vs. NC mimic)	*p* value (miR-505-MB vs. miR-505 mimic)
G1	46.36	57.15	66.34	0.0281	0.0117	0.0352
S	22.15	14.27	6.5	0.0341	0.0123	0.0368
G2	31.49	28.58	27.16	0.5785	0.3112	0.8749

miR-505: microRNA-505; NC: negative control; MB: microbubbles.

**Table 3:** The relationship between the AKT2 expression and cell cycle changes.

Cell cycle	miR-505-MB+OE-NC	miR-505-MB+OE-AKT2	*p* value
G1	66.15	51.75	0.0135
S	9.48	19.51	0.0174
G2	24.37	28.92	0.3965

miR-505: microRNA-505; NC: negative control; OE: overexpression.

## Data Availability

The data used to support the findings of this study are included in the article.
